# Ethyl 2-(2,3,4,5,6-Penta­bromo­phen­yl)acetate

**DOI:** 10.1107/S1600536810025626

**Published:** 2010-07-14

**Authors:** Anne M. Sauer, Art G. Mack, Hassan Y. Elnagar, Frank R. Fronczek

**Affiliations:** a Albemarle Process Development Center, Albemarle Corporation, PO Box 341, Baton Rouge, LA 70821, USA; b Department of Chemistry, Louisiana State University, Baton Rouge, LA 70803-1804, USA

## Abstract

The title compound PBPEA, C_10_H_7_Br_5_O_2_, has its ethyl acetate portion nearly orthogonal to the benzene ring, with a C—C—C—C torsion angle of 88.3 (5)°. The packing involves an inter­molecular contact with a Br⋯Br distance of 3.491 (1) Å, having C—Br⋯Br angles of 173.4 (2) and 106.0 (2)°. The crystal studied was an inversion twin.

## Related literature

For synthetic procedures, see: Holmes & Lightner (1995 ▶); Adams & Thal (1941 ▶). For a description of the Cambridge Structural Database, see: Allen (2002 ▶). For related structures, see: Eriksson & Hu (2002*a* ▶,*b* ▶); Eriksson *et al.* (1999 ▶); Köppen *et al.* (2007 ▶); Krigbaum & Wildman (1971 ▶); Mrse *et al.* (2000 ▶); Pedireddi *et al.* (1994 ▶); Williams *et al.* (1985 ▶).
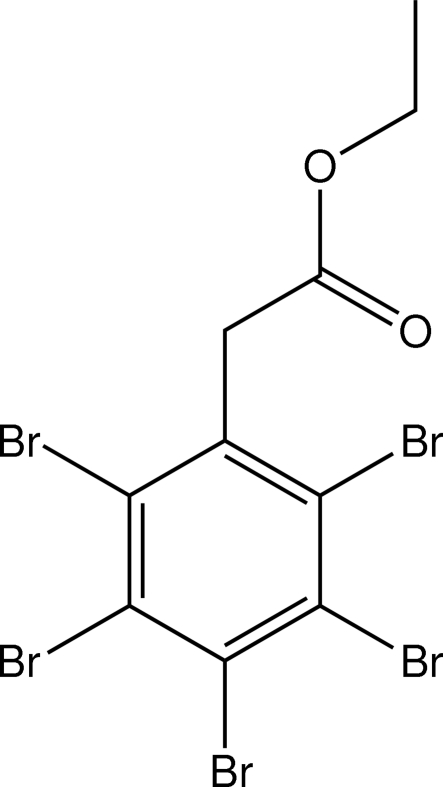


## Experimental

### 

#### Crystal data

C_10_H_7_Br_5_O_2_
*M* *_r_* = 558.71
Monoclinic, 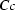

*a* = 4.6136 (10) Å
*b* = 22.548 (5) Å
*c* = 13.195 (2) Å
β = 90.993 (11)°
*V* = 1372.4 (5) Å^3^
*Z* = 4
Mo *K*α radiation
μ = 14.63 mm^−1^
*T* = 90 K
0.25 × 0.12 × 0.12 mm

#### Data collection

Nonius KappaCCD diffractometer with Oxford Cryostream
Absorption correction: multi-scan (*SCALEPACK*; Otwinowski & Minor, 1997 ▶) *T*_min_ = 0.121, *T*_max_ = 0.273
10525 measured reflections
3863 independent reflections
3676 reflections with *I* > 2σ(*I*)
*R*_int_ = 0.013

#### Refinement

*R*[*F* ^2^ > 2σ(*F* ^2^)] = 0.025
*wR*(*F* ^2^) = 0.053
*S* = 1.17
3863 reflections
157 parameters
2 restraints
H-atom parameters constrained
Δρ_max_ = 0.65 e Å^−3^
Δρ_min_ = −0.66 e Å^−3^
Absolute structure: Flack (1983 ▶), 1846 Friedel pairs
Flack parameter: 0.467 (13)

### 

Data collection: *COLLECT* (Nonius, 2000 ▶); cell refinement: *SCALEPACK* (Otwinowski & Minor, 1997 ▶); data reduction: *DENZO* (Otwinowski & Minor, 1997 ▶) and *SCALEPACK*; program(s) used to solve structure: *SIR97* (Altomare *et al.*, 1999 ▶); program(s) used to refine structure: *SHELXL97* (Sheldrick, 2008 ▶); molecular graphics: *ORTEP-3 for Windows* (Farrugia, 1997 ▶); software used to prepare material for publication: *SHELXL97*.

## Supplementary Material

Crystal structure: contains datablocks global, I. DOI: 10.1107/S1600536810025626/jj2038sup1.cif

Structure factors: contains datablocks I. DOI: 10.1107/S1600536810025626/jj2038Isup2.hkl

Additional supplementary materials:  crystallographic information; 3D view; checkCIF report

## Figures and Tables

**Fig. 1.**
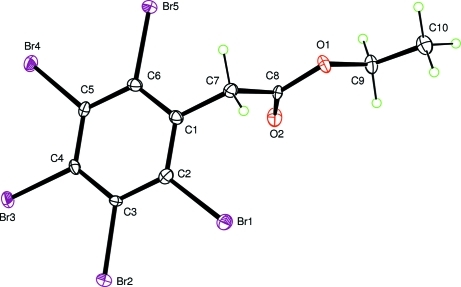
Ellipsoids at the 50% probability level, with H atoms having arbitrary radius.

**Fig. 2.**
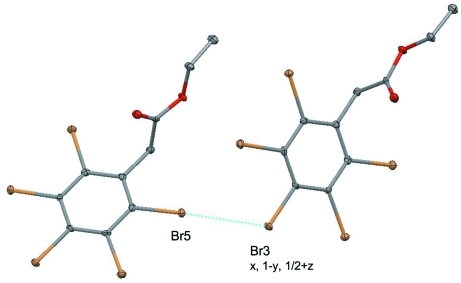
The intermolecular Br···Br contact. H atoms are omitted.

**Fig. 3.**
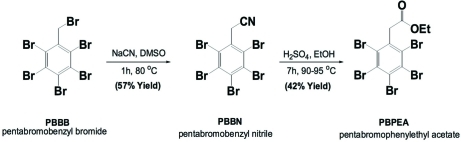
Preparation of the title compound.

## supplementary crystallographic information

### Comment

In an effort to prepare a series of proposed pentabromophenyl-substituted compounds necessary as analytical standards, the title ethyl ester derivative rendered itself to be an important intermediate and was synthesized *via* PBBN as an intermediate. This PBBN nitrile precursor was prepared by known procedures (Holmes & Lightner, 1995) from hexabromotoluene, henceforth referred to as pentabromobenzyl bromide, PBBB. Subsequent conversion of the resulting pentabromobenzyl nitrile intermediate to PBPEA was completed with ethanol in sulfuric acid. (Adams & Thal, 1941). The nature of such sterically hindered and electronically deprived pentabromo-compounds has provided a unique opportunity to examine the reactivity and resulting isolation / purification tendencies associated with these systems.

The ethyl acetate portion of the molecule (Fig. 1) is extended, with torsion angles C1—C7—C8—O1 174.8 (3)°, C7—C8—O1—C9 179.3 (3)°, C8—O1—C9—C10 - 165.1 (3)°, and it is nearly orthogonal to the phenyl ring, with C2—C1—C7—C8 torsion angle 88.3 (5)°. The C—Br distances are in the range 1.876 (4)–1.896 (4) Å, with mean value 1.887 Å. This value compares favorably with the mean value of 1.880 Å in decabromodiphenylethane (Köppen *et al.*, 2007), the only ordered entry in the CSD (version 5.31, Nov. 2009; Allen 2002) with Br_5_Ph on an *sp*^3^ C atom. The structure of pentabromotoluene has also been reported (Krigbaum & Wildman, 1971), but it has the methyl group statistically disordered, sharing all six sites with Br. Structures of several pentabromophenyl ethers have also been reported (Eriksson & Hu, 2002*a*,b; Eriksson *et al.*, 1999; Mrse *et al.*, 2000; Williams *et al.*, 1985), and the geometries of their Br_5_Ph groups are similar.

Packing of compounds containing Br_5_Ph groups usually involves intermolecular Br···Br contacts, and one such interaction exists in the structure of the title compound, as illustrated in Fig. 2. The contact is between glide-related molecules, and has Br3···Br5 distance 3.491 (1) Å. The angular disposition of the contact is termed type II by Pedireddi *et al.* (1994), having one C–Br···Br angle near linear and the other nearly orthogonal.  In this case, the angle about Br5 is 173.4 (2)°, and the angle about Br3 is 106.0 (2)°. Also, both O atoms make intermolecular contacts with Br, O1···Br4(1 + *x*, 1 - *y*, 1/2 + *z*) 3.184 (3) Å; O2···Br2 (*x* - 1/2, 3/2 - *y*, 1/2 + *z*) 3.123 (3) Å.

### Experimental

Preparation of PBBN (9263–183):(Fig. 3) To a 3-neck, 100-ml RBF, fitted with a nitrogen inlet, thermocouple and septum, was charged the starting PBBB (5 g, 8.84 mmol) in DMSO (50 ml). To this slurry was added the sodium cyanide (0.44 g, 8.98 mmol) in one portion at room temperature and the reaction mixture immediately became mint in color. This color quickly dissipated and became brown. The reaction was allowed to heat for one hour, with vigorous stirring, at 80 °C under an inert atmosphere. Upon conclusion, the contents were filtered hot to remove an insoluble material (1.01 g) and the resulting brown filtrate was treated with water to precipitate the PBBN product. The light brown solids (fluffy) were collected *via* suction filtration. Drying overnight afforded a dark brown solid. Solids were rinsed with IPA and filtered to provide 2.58 g PBBN material (light brown in color and free flowing) upon drying (~57% yield), mp = 178.6 & 179.5 °C. Purity of the crude PBBN was found to be ~70% (trimethylbenzene as internal standard) and was used without further purification. The trace unreacted sodium cyanide was destroyed by bleach solution in the aqueous DMSO solution.

Preparation of PBPEA (9263–189): (Fig. 3) To a 3-neck, 100-ml RBF, fitted with a reflux condenser, thermocouple, and nitrogen inlet was charged absolute ethanol (30 g). Concentrated sulfuric acid (30 g) as added slowly as to minimize exotherm. When heating subsided, the starting nitrile, PBBN (1.0 g), was added in one portion. The temperature was set to ~96 °C, and the contents were allowed to reflux for 7 h. After heating for ~15 minutes, the reaction turned dark brown in color with no visible evidence of insoluble PBBN. After 2 h. heating, reflux had stabilized. Gradually, the temperature dropped to ~88 °C. The reactor was cooled, and the contents were poured into ice water. Immediately, a grey-brown solid precipitate was formed and subsequently collected *via* suction filtration. Air-drying overnight provided 1.65 grams crude material. The solids were slurried in acetone and filtered to collect 0.46 grams (42.2% yield) brown solid on drying. Crude NMR revealed desired ethyl ester as the major component. ^1^H NMR: (400 MHz, DMSO-d6): δ = 4.32 (singlet, benzylic –CH_2_–, 2H), 4.17–4.12 (quartet, ester methylene, 2H), 1.22–1.19 (triplet, ester methyl, 3H). (Impurities consist of the acetic acid derivative, along with the amide intermediate.) Recrystallization from acetone / IPA afforded the title ester compound obtained in pure form as spear-like needles, mp (DSC-melt) = 142.9–145.8 °C.

### Refinement

H atoms on C were placed in idealized positions with C—H distances 0.98–0.99 Å and thereafter treated as riding. A torsional parameter was refined for the methyl group. *U*_iso_ for H were assigned as 1.2 times *U*_eq_ of the attached atoms (1.5 for methyl). The Flack (1983) parameter refined to a value of 0.467 (13), indicating a nearly perfect inversion twin. Friedel pairs were kept separate in the refinement.

### Crystal data

**Table:**

C_10_H_7_Br_5_O_2_	*F*(000) = 1032
*M**_r_* = 558.71	*D*_x_ = 2.704 Mg m^−^^3^
Monoclinic, *C**c*	Mo *K*α radiation, λ = 0.71073 Å
Hall symbol: C -2yc	Cell parameters from 2027 reflections
*a* = 4.6136 (10) Å	θ = 2.5–30.0°
*b* = 22.548 (5) Å	µ = 14.63 mm^−^^1^
*c* = 13.195 (2) Å	*T* = 90 K
β = 90.993 (11)°	Needle fragment, light brown
*V* = 1372.4 (5) Å^3^	0.25 × 0.12 × 0.12 mm
*Z* = 4	

### Data collection

**Table:**

Nonius KappaCCD diffractometer with Oxford Cryostream	3863 independent reflections
Radiation source: fine-focus sealed tube	3676 reflections with *I* > 2σ(*I*)
graphite	*R*_int_ = 0.013
ω and φ scans	θ_max_ = 30.0°, θ_min_ = 3.0°
Absorption correction: multi-scan (*SCALEPACK*; Otwinowski & Minor, 1997)	*h* = −6→6
*T*_min_ = 0.121, *T*_max_ = 0.273	*k* = −31→31
10525 measured reflections	*l* = −18→18

### Refinement

**Table:**

Refinement on *F*^2^	Hydrogen site location: inferred from neighbouring sites
Least-squares matrix: full	H-atom parameters constrained
*R*[*F*^2^ > 2σ(*F*^2^)] = 0.025	*w* = 1/[σ^2^(*F*_o_^2^) + (0.0154*P*)^2^ + 2.9894*P*] where *P* = (*F*_o_^2^ + 2*F*_c_^2^)/3
*wR*(*F*^2^) = 0.053	(Δ/σ)_max_ = 0.002
*S* = 1.17	Δρ_max_ = 0.65 e Å^−^^3^
3863 reflections	Δρ_min_ = −0.66 e Å^−^^3^
157 parameters	Extinction correction: *SHELXL97* (Sheldrick, 2008), Fc^*^=kFc[1+0.001xFc^2^λ^3^/sin(2θ)]^-1/4^
2 restraints	Extinction coefficient: 0.00100 (7)
Primary atom site location: structure-invariant direct methods	Absolute structure: Flack (1983), 1842 Friedel pairs
Secondary atom site location: difference Fourier map	Flack parameter: 0.467 (13)

### Special details

**Table:**

Experimental. PBBN: ^1^H NMR: (400MHz, DMSO-d6): δ = 4.46 (singlet, benzylic –CH_2_–, 2H); ^13^C NMR: (125MHz, DMSO-d6): δ = 134.06, 130.18, 129.66, 127.90, 116.37, 31.29.PBPEA: ^1^H NMR: (400 MHz, CDCl_3_): δ = 4.36 (singlet, benzylic –CH_2_–, 2H), 4.26–4.20 (quartet, ester methylene, 2H), 1.32–1.28 (triplet, ester methyl 2H), 4.26–4.20 (quartet, ester methylene, 2H), 1.32–1.28 (triplet, ester methyl, 3H). ^13^C NMR: (100 MHz, CDCl_3_): δ = 168.79, 137.56, 129.37, 129.1, 128.55, 61.98, 47.94, 14.60.
Geometry. All e.s.d.'s (except the e.s.d. in the dihedral angle between two l.s. planes) are estimated using the full covariance matrix. The cell e.s.d.'s are taken into account individually in the estimation of e.s.d.'s in distances, angles and torsion angles; correlations between e.s.d.'s in cell parameters are only used when they are defined by crystal symmetry. An approximate (isotropic) treatment of cell e.s.d.'s is used for estimating e.s.d.'s involving l.s. planes.
Refinement. Refinement of *F*^2^ against ALL reflections. The weighted *R*-factor *wR* and goodness of fit *S* are based on *F*^2^, conventional *R*-factors *R* are based on *F*, with *F* set to zero for negative *F*^2^. The threshold expression of *F*^2^ > σ(*F*^2^) is used only for calculating *R*-factors(gt) *etc*. and is not relevant to the choice of reflections for refinement. *R*-factors based on *F*^2^ are statistically about twice as large as those based on *F*, and *R*- factors based on ALL data will be even larger.

### Fractional atomic coordinates and isotropic or equivalent isotropic displacement parameters (Å^2^)

**Table:**

	*x*	*y*	*z*	*U*_iso_*/*U*_eq_	
Br1	0.88580 (8)	0.721320 (18)	0.44969 (3)	0.01668 (9)	
Br2	0.57652 (8)	0.735821 (17)	0.22457 (3)	0.01500 (9)	
Br3	0.16294 (9)	0.628526 (18)	0.13378 (3)	0.01501 (9)	
Br4	0.06205 (8)	0.507432 (18)	0.26957 (3)	0.01482 (9)	
Br5	0.36995 (8)	0.495585 (18)	0.49436 (3)	0.01383 (9)	
O1	0.7331 (6)	0.61466 (13)	0.7294 (2)	0.0136 (6)	
O2	0.3795 (6)	0.65392 (14)	0.6300 (2)	0.0162 (6)	
C1	0.6196 (9)	0.60801 (18)	0.4523 (3)	0.0113 (7)	
C2	0.6548 (8)	0.65956 (18)	0.3941 (3)	0.0115 (8)	
C3	0.5193 (9)	0.66594 (17)	0.3000 (3)	0.0096 (7)	
C4	0.3429 (8)	0.62048 (18)	0.2616 (3)	0.0103 (7)	
C5	0.2983 (8)	0.56956 (18)	0.3187 (3)	0.0110 (8)	
C6	0.4373 (8)	0.56364 (18)	0.4137 (3)	0.0118 (8)	
C7	0.7750 (9)	0.60088 (18)	0.5534 (3)	0.0122 (8)	
H7A	0.9647	0.6213	0.5510	0.015*	
H7B	0.8118	0.5582	0.5660	0.015*	
C8	0.6017 (8)	0.62599 (17)	0.6398 (3)	0.0101 (7)	
C9	0.5844 (9)	0.6377 (2)	0.8175 (3)	0.0148 (8)	
H9A	0.4116	0.6132	0.8316	0.018*	
H9B	0.5200	0.6790	0.8046	0.018*	
C10	0.7928 (10)	0.6360 (2)	0.9071 (3)	0.0170 (9)	
H10A	0.8614	0.5953	0.9176	0.026*	
H10B	0.6938	0.6497	0.9679	0.026*	
H10C	0.9583	0.6620	0.8939	0.026*	

### Atomic displacement parameters (Å^2^)

**Table:**

	*U*^11^	*U*^22^	*U*^33^	*U*^12^	*U*^13^	*U*^23^
Br1	0.0201 (2)	0.01398 (19)	0.0157 (2)	−0.00486 (17)	−0.00499 (16)	0.00049 (16)
Br2	0.0221 (2)	0.01098 (19)	0.01185 (18)	−0.00063 (16)	−0.00041 (15)	0.00243 (15)
Br3	0.0200 (2)	0.01540 (18)	0.00946 (16)	0.00203 (16)	−0.00374 (14)	−0.00085 (16)
Br4	0.01717 (19)	0.0141 (2)	0.0131 (2)	−0.00421 (16)	−0.00080 (16)	−0.00249 (16)
Br5	0.01896 (19)	0.01114 (19)	0.0114 (2)	−0.00113 (16)	0.00103 (16)	0.00194 (15)
O1	0.0148 (13)	0.0184 (15)	0.0076 (12)	0.0050 (11)	−0.0008 (11)	−0.0002 (11)
O2	0.0156 (14)	0.0184 (15)	0.0144 (13)	0.0054 (12)	−0.0028 (11)	−0.0036 (12)
C1	0.0129 (17)	0.0116 (19)	0.0095 (17)	0.0013 (14)	0.0024 (15)	0.0005 (14)
C2	0.0104 (18)	0.0118 (19)	0.0125 (18)	−0.0011 (14)	0.0040 (15)	−0.0021 (14)
C3	0.0133 (17)	0.0070 (18)	0.0086 (16)	0.0010 (14)	0.0003 (14)	0.0017 (13)
C4	0.0103 (17)	0.0134 (19)	0.0072 (16)	0.0023 (14)	−0.0022 (14)	−0.0004 (14)
C5	0.0112 (18)	0.0114 (19)	0.0105 (18)	−0.0019 (14)	−0.0001 (14)	−0.0039 (14)
C6	0.0149 (19)	0.0101 (19)	0.0106 (18)	0.0026 (15)	0.0040 (15)	0.0014 (14)
C7	0.0127 (18)	0.0109 (18)	0.0129 (19)	−0.0013 (14)	−0.0018 (15)	0.0000 (15)
C8	0.0121 (18)	0.0098 (17)	0.0081 (16)	−0.0021 (14)	−0.0031 (14)	−0.0023 (14)
C9	0.015 (2)	0.019 (2)	0.0101 (18)	0.0031 (16)	−0.0008 (15)	−0.0034 (16)
C10	0.016 (2)	0.022 (2)	0.0139 (19)	0.0032 (17)	−0.0003 (16)	−0.0005 (16)

### Geometric parameters (Å, °)

**Table:**

Br1—C2	1.894 (4)	C3—C4	1.398 (6)
Br2—C3	1.885 (4)	C4—C5	1.391 (6)
Br3—C4	1.876 (4)	C5—C6	1.404 (5)
Br4—C5	1.883 (4)	C7—C8	1.514 (5)
Br5—C6	1.896 (4)	C7—H7A	0.9900
O1—C8	1.344 (5)	C7—H7B	0.9900
O1—C9	1.456 (5)	C9—C10	1.511 (6)
O2—C8	1.208 (5)	C9—H9A	0.9900
C1—C6	1.397 (6)	C9—H9B	0.9900
C1—C2	1.404 (5)	C10—H10A	0.9800
C1—C7	1.512 (5)	C10—H10B	0.9800
C2—C3	1.388 (6)	C10—H10C	0.9800
			
C8—O1—C9	115.0 (3)	C1—C7—H7A	109.2
C6—C1—C2	117.9 (4)	C8—C7—H7A	109.2
C6—C1—C7	121.2 (4)	C1—C7—H7B	109.2
C2—C1—C7	120.9 (4)	C8—C7—H7B	109.2
C3—C2—C1	121.4 (4)	H7A—C7—H7B	107.9
C3—C2—Br1	120.8 (3)	O2—C8—O1	124.2 (4)
C1—C2—Br1	117.8 (3)	O2—C8—C7	124.9 (4)
C2—C3—C4	119.9 (4)	O1—C8—C7	110.8 (3)
C2—C3—Br2	119.6 (3)	O1—C9—C10	108.3 (3)
C4—C3—Br2	120.5 (3)	O1—C9—H9A	110.0
C5—C4—C3	120.0 (3)	C10—C9—H9A	110.0
C5—C4—Br3	120.0 (3)	O1—C9—H9B	110.0
C3—C4—Br3	120.0 (3)	C10—C9—H9B	110.0
C4—C5—C6	119.5 (4)	H9A—C9—H9B	108.4
C4—C5—Br4	121.2 (3)	C9—C10—H10A	109.5
C6—C5—Br4	119.3 (3)	C9—C10—H10B	109.5
C1—C6—C5	121.3 (4)	H10A—C10—H10B	109.5
C1—C6—Br5	118.6 (3)	C9—C10—H10C	109.5
C5—C6—Br5	120.1 (3)	H10A—C10—H10C	109.5
C1—C7—C8	112.1 (3)	H10B—C10—H10C	109.5
			
C6—C1—C2—C3	−1.6 (6)	C2—C1—C6—C5	1.5 (6)
C7—C1—C2—C3	178.3 (4)	C7—C1—C6—C5	−178.5 (4)
C6—C1—C2—Br1	176.8 (3)	C2—C1—C6—Br5	−176.6 (3)
C7—C1—C2—Br1	−3.3 (5)	C7—C1—C6—Br5	3.5 (5)
C1—C2—C3—C4	0.2 (6)	C4—C5—C6—C1	0.1 (6)
Br1—C2—C3—C4	−178.1 (3)	Br4—C5—C6—C1	178.6 (3)
C1—C2—C3—Br2	−179.5 (3)	C4—C5—C6—Br5	178.1 (3)
Br1—C2—C3—Br2	2.2 (5)	Br4—C5—C6—Br5	−3.4 (4)
C2—C3—C4—C5	1.5 (6)	C6—C1—C7—C8	−91.8 (5)
Br2—C3—C4—C5	−178.9 (3)	C2—C1—C7—C8	88.3 (5)
C2—C3—C4—Br3	−179.3 (3)	C9—O1—C8—O2	1.2 (6)
Br2—C3—C4—Br3	0.4 (5)	C9—O1—C8—C7	179.3 (3)
C3—C4—C5—C6	−1.6 (6)	C1—C7—C8—O2	−7.1 (6)
Br3—C4—C5—C6	179.1 (3)	C1—C7—C8—O1	174.8 (3)
C3—C4—C5—Br4	180.0 (3)	C8—O1—C9—C10	−165.1 (3)
Br3—C4—C5—Br4	0.7 (5)		
